# Detection of Atrial Fibrillation Using Insertable Cardiac Monitors in Patients With Cryptogenic Stroke in Japan (the LOOK Study): Protocol for a Prospective Multicenter Observational Study

**DOI:** 10.2196/39307

**Published:** 2023-04-13

**Authors:** Satoshi Suda, Takehiro Katano, Kazuo Kitagawa, Yasuyuki Iguchi, Shigeru Fujimoto, Kenjiro Ono, Osamu Kano, Hidehiro Takekawa, Masatoshi Koga, Masafumi Ihara, Masafumi Morimoto, Hiroshi Yamagami, Tadashi Terasaki, Keiji Yamaguchi, Seiji Okubo, Yuji Ueno, Nobuyuki Ohara, Yuki Kamiya, Masataka Takeuchi, Yukako Yazawa, Yuka Terasawa, Ryosuke Doijiri, Yoshifumi Tsuboi, Kazutaka Sonoda, Koichi Nomura, Takashi Shimoyama, Akihito Kutsuna, Kazumi Kimura

**Affiliations:** 1 Department of Neurology, Nippon Medical School Tokyo Japan; 2 Department of Neurology, Tokyo Women's Medical University Tokyo Japan; 3 Department of Neurology, The Jikei University School of Medicine Tokyo Japan; 4 Division of Neurology, Department of Medicine, Jichi Medical University Tochigi Japan; 5 Division of Neurology, Department of Internal Medicine, Showa University School of Medicine Tokyo Japan; 6 Department of Neurology, Toho University Faculty of Medicine Tokyo Japan; 7 Department of Neurology, Dokkyo Medical University Tochigi Japan; 8 Department of Cerebrovascular Medicine, National Cerebral and Cardiovascular Center Osaka Japan; 9 Department of Neurology, National Cerebral and Cardiovascular Center Osaka Japan; 10 Department of Neurosurgery, Yokohamashintoshi Neurosurgical Hospital Kanagawa Japan; 11 Department of Stroke Neurology, National Hospital Organization Osaka National Hospital Osaka Japan; 12 Department of Neurology, Japanese Red Cross Kumamoto Hospital Kumamoto Japan; 13 Department of Neurology, Ichinomiya Nishi Hospital Aichi Japan; 14 Department of Cerebrovascular Medicine, NTT Medical Center Tokyo Tokyo Japan; 15 Department of Neurology, Juntendo University Faculty of Medicine Tokyo Japan; 16 Department of Neurology, Kobe City Medical Center General Hospital Hyogo Japan; 17 Department of Neurology, Showa University Koto Toyosu Hospital Tokyo Japan; 18 Department of Neurosurgery, Seisho Hospital Kanagawa Japan; 19 Department of Stroke Neurology, Kohnan Hospital Miyagi Japan; 20 Department of Neurology, Brain Attack Center Ota Memorial Hospital Hiroshima Japan; 21 Department of Neurology, Iwate Prefectural Central Hospital Iwate Japan; 22 Department of Neurosurgery, Kawasakisaiwai Hospital Kanagawa Japan; 23 Department of Neurology, Saiseikai Fukuoka General Hospital Fukuoka Japan; 24 Department of Neurology, Shioda Hospital Chiba Japan; 25 Department of Neurology, New Tokyo Hospital Chiba Japan

**Keywords:** atrial cardiomyopathy, atrial fibrillation, biomarker, cryptogenic stroke, insertable cardiac monitor

## Abstract

**Background:**

Paroxysmal atrial fibrillation (AF) is a probable cause of cryptogenic stroke (CS), and its detection and treatment are important for the secondary prevention of stroke. Insertable cardiac monitors (ICMs) are clinically effective in screening for AF and are superior to conventional short-term cardiac monitoring. Japanese guidelines for determining clinical indications for ICMs in CS are stricter than those in Western countries. Differences between Japanese and Western guidelines may impact the detection rate and prediction of AF via ICMs in patients with CS. Available data on Japanese patients are limited to small retrospective studies. Furthermore, additional information about AF detection, including the number of episodes, cumulative episode duration, anticoagulation initiation (type and dose of regimen and time of initiation), rate of catheter ablation, role of atrial cardiomyopathy, and stroke recurrence (time of recurrence and cause of the recurrent event), was not provided in the vast majority of previously published studies.

**Objective:**

In this study, we aim to identify the proportion and timing of AF detection and risk stratification criteria in patients with CS in real-world settings in Japan.

**Methods:**

This is a multicenter, prospective, observational study that aims to use ICMs to evaluate the proportion, timing, and characteristics of AF detection in patients diagnosed with CS. We will investigate the first detection of AF within the initial 6, 12, and 24 months of follow-up after ICM implantation. Patient characteristics, laboratory data, atrial cardiomyopathy markers, serial magnetic resonance imaging findings at baseline, 6, 12, and 24 months after ICM implantation, electrocardiogram readings, transesophageal echocardiography findings, cognitive status, stroke recurrence, and functional outcomes will be compared between patients with AF and patients without AF. Furthermore, we will obtain additional information regarding the number of AF episodes, duration of cumulative AF episodes, and time of anticoagulation initiation.

**Results:**

Study recruitment began in February 2020, and thus far, 213 patients have provided written informed consent and are currently in the follow-up phase. The last recruited participant (May 2021) will have completed the 24-month follow-up in May 2023. The main results are expected to be submitted for publication in 2023.

**Conclusions:**

The findings of this study will help identify AF markers and generate a risk scoring system with a novel and superior screening algorithm for occult AF detection while identifying candidates for ICM implantation and aiding the development of diagnostic criteria for CS in Japan.

**Trial Registration:**

UMIN Clinical Trial Registry UMIN000039809; https://tinyurl.com/3jaewe6a

**International Registered Report Identifier (IRRID):**

DERR1-10.2196/39307

## Introduction

Stroke is a leading cause of mortality and disability worldwide. A primary goal of stroke management is to clarify the stroke etiology to optimize secondary prevention. Despite standard extensive diagnostic workups, approximately 15%-40% of all ischemic stroke cases are of unknown etiologies and are classified as cryptogenic [1]. Cryptogenic stroke (CS) is heterogeneous in origin, but most cases involve embolic mechanisms. Paroxysmal atrial ﬁbrillation (AF) is suspected to be a major cause of CS, and patients are likely to develop severe cerebral infarction in the absence of anticoagulation therapy. Current guidelines recommend a secondary prevention regimen of antiplatelet agents to prevent stroke recurrence in patients diagnosed with CS and for whom the AF status is unknown. Long-term cardiac rhythm monitoring can reveal occult AF that may otherwise be missed by conventional short-term monitoring [2]. Since September 2016, insertable cardiac monitors (ICMs) for patients with CS have been covered by insurance in Japan.

The term “embolic stroke of undetermined source” (ESUS) has been proposed to describe CS that is not lacunar and is not associated with proximal arterial stenosis or a recognizable cardioembolic source [1]. Two international ESUS trials demonstrated that oral anticoagulants (rivaroxaban and dabigatran) were not superior to aspirin for the prevention of recurrent stroke after ESUS [3,4], although a secondary analysis in the RE-SPECT ESUS (Randomized, Double-Blind Evaluation in Secondary Stroke Prevention Comparing the Efficacy and Safety of the Oral Thrombin Inhibitor Dabigatran Etexilate Versus Acetylsalicylic Acid in Patients With Embolic Stroke of Undetermined Source) study demonstrated that, compared with aspirin, dabigatran was associated with a lower risk of recurrent stroke among Japanese patients with ESUS [5]. In Japan, magnetic resonance imaging (MRI) is widely used in the diagnosis of stroke. The Japanese guidelines indicate that MRI is essential in the selection of candidates with CS eligible for ICM implantation [6]. Branch atheromatous disease (BAD) is a single subcortical infarction larger than lacunar stroke in the territories of deep perforators without relevant arterial stenosis [7]. The Japanese guidelines on the clinical indications for ICMs recommend that BAD should not be considered as an indication because it results from a nonembolic mechanism [6]. These procedural differences between Japan and Western countries may impact the detection rate and predictors of AF in patients with CS. However, available data on Japanese patients are limited to small retrospective studies. Furthermore, additional information about AF detection, including the number of episodes, cumulative episode duration, anticoagulation initiation (type and dose of regimen and time of initiation), rate of catheter ablation, role of atrial cardiomyopathy, and stroke recurrence (time of recurrence and cause of the recurrent event), was not provided in the vast majority of previously published studies [8,9].

This study aims to clarify the proportion and timing of AF detection via ICMs in patients diagnosed with CS in Japan. Additionally, we aim to identify risk stratification criteria using real-world data, which could then be used in clinical practice to predict occult AF, identify optimal candidates for ICM implantation, and inform the development of diagnostic criteria.

## Methods

### Study Design

The LOOK (a multicenter observational study on the detection of AF using insertabLe cardiac mOnitors in patients with cryptOgenic stroKe) registry is an observational, multicenter, prospective registry of patients who have been diagnosed with CS and implanted with an ICM. The Medtronic CareLink Network is being used to remotely transmit the device data.

### Ethics Approval

Ethical approval for this study was obtained from the ethics review committee of Nippon Medical School (B-2019-043) and from the relevant ethics committees of all participating centers. Written informed consent is required from all patients or their family members before study participation. This study is registered with the University hospital Medical Information Network (UMIN) Clinical Trial Registry (UMIN000039809).

### Patient Population

Patient enrollment for this ongoing study started in February 2020 in 24 medical institutions throughout Japan. The inclusion criteria for this study are as follows: (1) patients who have been diagnosed with CS according to the Japanese diagnostic criteria and have been implanted with an ICM (Reveal LINQ), and (2) patients aged 40-90 years. The main exclusion criterion is the presence of contraindications for MRI. A full description of the inclusion and exclusion criteria is presented in Textbox 1.

**Inclusion and exclusion criteria.**

**Inclusion criteria**

- Patients of either sex, between 40 and 90 years of age
- Patients diagnosed with cryptogenic stroke and implanted with an insertable cardiac monitor
- Patients (or their legal representatives) who have given written consent

**Exclusion criteria**

- Patients with a history of atrial fibrillation and atrial flutter
- Patients with permanent contraindications or indications for oral anticoagulants
- Applicable patients with pacemaker or implantable defibrillator implantation
- Magnetic resonance imaging–contraindicated patients
- Patients diagnosed with life expectancy within 2 years due to some disease
- An inappropriate decision made by the investigator

### Data Collection

The study data, such as patient background, laboratory data, coagulation test, B-type natriuretic peptide, physiological examination (electrocardiogram [ECG], transthoracic echocardiography, transesophageal echocardiography [TEE], and carotid echocardiography), neuroimaging examination, Mini-Mental State Examination (MMSE), modified Rankin Scale (mRS), and presence of cardiovascular events, gastrointestinal bleeding, and other adverse events, or death (Table 1 and Textbox 2), will be stored on an encrypted website and will be accessible only to study researchers with appropriate training and ethics review board approval. Data monitoring will be performed independently by the study steering committee.

**Table 1:** Overview of study schedule.

Survey items	Baseline survey	6 months (±30 days)	12 months (±30 days)	24 months (±30 days)
Consent withdrawal/visit status/health confirmation		✔	✔	✔
Patient background/history	✔			
Blood count, biochemistry, coagulation test, BNP^a^	✔	✔	✔	✔
Physiological examination (ECG,^b^ TTE,^c^ TEE,^d^ carotid echocardiography)	✔			
Brain MRI^e^ examination	✔	✔	✔	✔
MMSE^f^	✔		✔	✔
mRS^g^	✔	✔	✔	✔
Presence of cardiovascular events, gastrointestinal bleeding, other adverse events, or death		✔	✔	✔

^a^BNP: B-type natriuretic peptide.

^b^ECG: electrocardiogram.

^c^TTE: transthoracic echocardiography.

^d^TEE: transesophageal echocardiography.

^e^MRI: magnetic resonance imaging.

^f^MMSE: Mini-Mental State Examination.

^g^mRS: modified Rankin Scale.

**Detailed survey items in this study.**

**Patient characteristics**

- Age, sex, body mass index, prestroke modified Rankin Scale (mRS) score, antithrombotic therapy before the index case, previous stroke, hypertension, dyslipidemia, diabetes mellitus, alcohol intake, smoking, peripheral artery disease, history of cancer, education level, and CHA_2_DS_2_-VASc (Congestive Heart Failure, Hypertension, Age [≥75 years], Diabetes, Stroke/Transient Ischemic Attack, Vascular Disease, Age [65-74 years], Sex [Female]) score

**Neuroimaging**

- Distribution of infarct lesions, infarct size, number of infarcts, major vessel occlusion, intra-arterial signal signs, susceptibility vessel signs, Diffusion-Weighted Imaging—Alberta Stroke Program Early Computed Tomography Scores, natural recanalization, hemorrhagic transformation, number and location of cerebral microbleeds, old cortical lesions, and recurrence of stroke after magnetic resonance imaging

**Electrocardiogram (ECG) and Holter ECG**

- Deep terminal negativity of the P-wave V1, P-wave terminal force in lead V1, P axis, PR interval, heart rate–corrected QT interval, and the number and percentage of atrial premature contractions and ventricular premature contractions

**Blood examination**

- Routine blood biochemistry examinations, including creatinine clearance and measurements of D-dimers, C-reactive protein, brain natriuretic peptide (BNP), and N-terminal prohormones of the BNP

**Transthoracic echocardiography and transesophageal echocardiography**

- Left atrium diameter, left atrial volume index, valvular disease, right to left shunt, aortic plaque thickness and morphology, spontaneous echo contrast, left atrial appendage filling and emptying velocity, left ventricular ejection fraction, atrial septal aneurysm, and strand readings

**Carotid ultrasound**

- Max intima-media thickness, plaque morphology, internal carotid artery stenosis as assessed by the European Carotid Surgery Trial method, peak systolic velocity, and end diastolic velocity

**Incidence of recurrent stroke or transient ischemic attack**

**Use and type of oral antithrombotic therapy and antiarrhythmic drugs**

**Stroke severity assessed using the mRS and the National Institutes of Health Stroke Scale**

**Cognitive status evaluated through the Mini-Mental State Examination**

**Cardiovascular events, gastrointestinal bleeding, cancer, other adverse events, or death**

### Sample Size

Recent retrospective observational ICM studies have demonstrated that major vessel occlusion (n=84) and frequent premature atrial contractions (n=66) were associated with AF detection after adjustment for risk factors and comorbidities, respectively [8,9]. A subanalysis of the CRYSTAL AF (Cryptogenic Stroke and Underlying AF) study (including 221 patients with a 30% AF detection rate) demonstrated that older age and longer PR interval were associated with an increased likelihood of detecting AF [10]. On the basis of the results of these previous studies, we decided to enroll 200 patients in this observational study to perform statistical analyses, including multivariate regression analysis, with sufficient statistical power.

### Data Analysis Plan

Table 1 presents an overview of the protocol schedule for this study. Categorical variables will be expressed as frequencies and percentages, whereas continuous variables will be expressed as mean with SD or median with IQR, as appropriate. Patient characteristics, medical history, vital signs, laboratory data, the CHA_2_DS_2_-VASc (Congestive Heart Failure, Hypertension, Age [≥75 years], Diabetes, Stroke/Transient Ischemic Attack, Vascular Disease, Age [65-74 years], Sex [Female]) score, neuroimaging characteristics, ECG characteristics, TEE characteristics, MMSE score, mRS score, and stroke recurrence will be compared between patients with AF and patients without AF (Textbox 2). Intergroup differences will be assessed using the chi-square test or the Wilcoxon rank-sum test, as appropriate. The Yates correction or the Fisher exact test will be used if the validity conditions for the chi-square test are not met (theoretical number <5). Furthermore, we will obtain additional information regarding the number of AF episodes, duration of cumulative AF episodes, and time of anticoagulation initiation. We will investigate the rate of initial AF detection within the initial 6, 12, and 24 months after ICM implantation using Kaplan-Meier survival estimates. Cox proportional hazards regression models will be fitted to various baseline characteristics for the prediction of AF. Receiver operating characteristic curve analysis will be performed to assess the predictive accuracy of a variable in AF detection. All analyses will be performed via JMP version 13 (SAS Institute Inc), with *P*<.05 indicating statistical significance.

### Study Organization

The LOOK study has been organized by a central coordinating center at the Department of Neurology, Nippon Medical School, and is being conducted at 24 medical centers throughout Japan. A steering committee is managing the trial.

## Results

Study recruitment started in February 2020, and thus far, 213 patients have provided written informed consent in May 2021 and are currently in the follow-up phase. The last recruited participant will have completed the 24-month follow-up in May 2023. Data cleanup and analyses are projected to be completed by September 2023, and the results are expected to be submitted for peer-reviewed publication in 2023.

## Discussion

### Principal Findings

Recent meta-analyses demonstrate that, compared with short-term monitoring, prolonged cardiac rhythm monitoring is associated with higher rates of AF detection, a higher incidence of anticoagulation initiation, and reduced stroke recurrence in patients with CS [11]. However, these studies were mainly conducted in Western countries, and the generalizability of these findings to Asian populations is unclear. We are therefore conducting this study, in which we will recruit patients prospectively and identify the proportion of patients with AF to effectively compare the characteristics and outcomes with respect to the presence and absence of AF in patients with CS in Japan.

Some testing biases are affected by geographic location; for example, brain MRI is used less frequently in European patients with ESUS [12]. However, there is better access to MRI in Japan, and it is often implemented upon hospital admission as well as during follow-ups. Furthermore, MRI is necessary for the selection of candidates for ICMs according to Japanese guidelines [6]. This study is thus investigating detailed MRI findings, such as the presence of lesion patterns, susceptibility vessel signs, hemorrhagic transformation, old cortical lesions, and new asymptomatic lesions, during follow-up.

Recent evidence indicates that atrial cardiomyopathy, diagnosed by the presence of serum or electrocardiographic biomarkers, leads to AF and may result in thromboembolism even before AF manifests [13,14]. In this study, we are investigating not only the left atrial diameter but also the left atrial volume index and P-wave parameters in ECG, including P-wave terminal force in V1. Atrial cardiomyopathy may explain a proportion of ESUS cases, although AF is currently the sole diagnostic criterion for atrial thrombogenic potential. ARCADIA (AtRial Cardiopathy and Antithrombotic Drugs In Prevention After Cryptogenic Stroke) is an ongoing trial investigating whether anticoagulant therapy reduces the occurrence of stroke in patients with atrial cardiomyopathy without known AF [15]. This study will clarify the association among atrial cardiomyopathy markers, AF, and stroke recurrence in patients with CS.

A previous retrospective study reported that plasma brain natriuretic peptide (BNP) levels were elevated in the acute phase of stroke but were substantially reduced in patients with cardioembolic stroke [16]. This suggests that fluctuating levels of BNP or N-terminal prohormone of BNP (NT-proBNP) may be associated with occult paroxysmal AF. However, serial changes in plasma BNP or NT-proBNP levels in patients with CS and ICMs have not been fully investigated. In this study, we will be conducting serial measurements of BNP or NT-proBNP levels.

### Conclusions

By conducting this prospective LOOK multicenter study, the characteristics of patients with occult AF detected by ICMs will be clarified in more detail than previously reported by analyzing imaging studies on admission and during follow-up, serial measurements of blood markers, and various physiological findings. We hope that our findings will contribute to the identification of markers and enable the development of a risk scoring system with a new and better screening algorithm, which could inform future guidelines for stroke screening in patients with CS as well as the development of comprehensive clinical trials.

## Abbreviations

AF: atrial fibrillation
BAD: branch atheromatous disease
BNP: brain natriuretic peptide
CS: cryptogenic stroke
ECG: electrocardiogram
ESUS: embolic stroke of undetermined source
ICM: insertable cardiac monitor
MMSE: Mini-Mental State Examination
MRI: magnetic resonance imaging
mRS: modified Rankin Scale
NT-proBNP: N-terminal prohormone of brain natriuretic peptide
TEE: transesophageal echocardiography

## Data Availability

Deidentified participant data will be shared on request. Researchers who make the request should include a methodologically sound proposal on how the data will be used. They will also need to sign a data access agreement. The corresponding author can be contacted directly to request data sharing.
